# Contemporary attitudes and beliefs on coronary artery calcium from social media using artificial intelligence

**DOI:** 10.1038/s41746-024-01077-w

**Published:** 2024-03-30

**Authors:** Sulaiman Somani, Sujana Balla, Allison W. Peng, Ramzi Dudum, Sneha Jain, Khurram Nasir, David J. Maron, Tina Hernandez-Boussard, Fatima Rodriguez

**Affiliations:** 1 Department of Medicine, Stanford University, Stanford, CA USA; 2 Cardiovascular Institute, Stanford University, Stanford, CA USA; 3 Department of Medicine, University of California, San Francisco—Fresno, Fresno, CA USA; 4 Division of Cardiovascular Medicine, Stanford University, Stanford, CA USA; 5 Division of Cardiovascular Prevention and Wellness, Department of Cardiology, Houston Methodist DeBakey Heart & Vascular Center, Houston, TX USA; 6 Stanford Prevention Research Center, Palo Alto, CA USA; 7 Department of Biomedical Data Science, Stanford University, Stanford, CA USA; 8 Center for Digital Health, Stanford University, CA, USA

**Keywords:** Preventive medicine, Machine learning, Data mining, Cardiovascular diseases, Risk factors

## Abstract

Coronary artery calcium (CAC) is a powerful tool to refine atherosclerotic cardiovascular disease (ASCVD) risk assessment. Despite its growing interest, contemporary public attitudes around CAC are not well-described in literature and have important implications for shared decision-making around cardiovascular prevention. We used an artificial intelligence (AI) pipeline consisting of a semi-supervised natural language processing model and unsupervised machine learning techniques to analyze 5,606 CAC-related discussions on Reddit. A total of 91 discussion topics were identified and were classified into 14 overarching thematic groups. These included the strong impact of CAC on therapeutic decision-making, ongoing non-evidence-based use of CAC testing, and the patient perceived downsides of CAC testing (e.g., radiation risk). Sentiment analysis also revealed that most discussions had a neutral (49.5%) or negative (48.4%) sentiment. The results of this study demonstrate the potential of an AI-based approach to analyze large, publicly available social media data to generate insights into public perceptions about CAC, which may help guide strategies to improve shared decision-making around ASCVD management and public health interventions.

Atherosclerotic cardiovascular disease (ASCVD) remains the leading cause of death in the United States^1^. Earlier identification and intervention of ASCVD is critical for reducing its morbidity and mortality, as over a third of all ASCVD deaths occur in individuals with no prior symptoms^1^. Detection of coronary artery calcification (CAC) by a specialized computed tomography (CT) scan (“CAC scan”) can help guide patient and clinicians on shared-decision making around cardiovascular risk assessment^2^. As such, CAC scans are endorsed by multiple medical societies as power tools for personalizing cardiovascular risk and preventive therapy recommendations^3,4^. CAC may also be a strong motivator for improving health behaviors, including lifestyle changes and adherence to preventive therapies like statins^5,6^.

While public interest in CAC has grown over time, current public perceptions about CAC are not well-described^7^. Understanding these beliefs about CAC is critical, as it may help frame shared decision-making discussions and guide public health interventions around ASCVD. Artificial intelligence (AI)-enabled analysis of large volumes of social media data can provide an efficient approach for analyzing contemporary public opinions on common health-related topics and allow for a systematic evaluation of emerging themes^8^. Reddit is a free and widely used social media platform with over 52 million daily active users and over 30 billion views every month^9^. In this study, we leverage an artificial intelligence pipeline using natural language processing and unsupervised learning to characterize real-world perceptions about CAC using discussions on Reddit.

We extracted a total of 5606 unique CAC-related discussions (1017 posts, 4589 comments) from 3545 unique users across 990 subreddits from March 29, 2008, through May 21, 2023 (Supplementary Fig. 1). The largest number of discussions from a single author was 26, while 3463 (97.7%) authors contributed less than six discussions each. The subreddits with the most discussions were r/keto (7.5%), r/Cholesterol (7.0%), and r/AskDocs (5.8% of all discussions). The number of CAC-related discussions increased by an average of 57.2% yearly. Using a pretrained, sentence-level Bidirectional Encoder Representations from Transformers (BERT) model, we embedded these discussions into a vectorized language space, in which they were further dimensionally reduced and clustered to identify a total of 91 topics (Fig. 1). These topics were further clustered to identify 14 overarching groups. The largest topics and groups centered around CAC testing to evaluate symptoms (e.g., palpitations, chest pain, and anxiety) and de-risking non-ischemic cardiovascular disease (groups 1, 5); interpreting CAC scores in the context of lifestyle and lipid results (groups 2, 4, 8, and 10); and the disadvantages of CAC testing (e.g., financial cost, radiation risk) (Table 1). Other notable topics included indications for CAC testing (e.g., topics 10, 27, 42, 48), CAC and statins (e.g., topics 24, 31, 34), ketogenic diets can affect CAC (e.g., topics 16, 19), radiation exposure risk (e.g., topics 22, 45), insurance issues (e.g., topics 29, 30, 37, 50), and celebrities with CAC (e.g., topics 43, 55, 56). A separate pretrained BERT model was used to analyze the sentiment of each discussion, uncovering that 49.5% of discussions were neutral, 48.4% were negative, and 2.1% were positive. The average sentiment of all discussions remained stably neutral-to-negative (−0.42 – −0.50) each year from 2013 through 2023 (Supplementary Fig. 2).

**Fig. 1:**
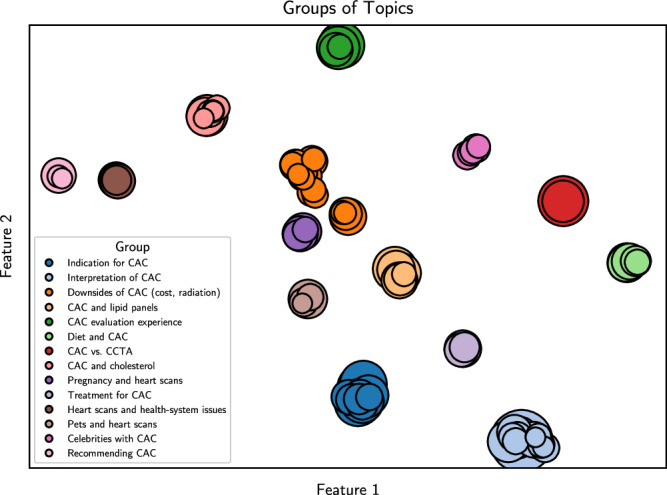
Topic modeling revealed 91 topics and 14 groups. Visual representation of the topics (circles) and groups (color); the size of each topic (circle) represents the relative number of discussions grouped in that topic. The *x*- and *y*-axes represent the two Uniform Manifold Approximation and Projection features onto which topics were dimensionally reduced to allow their visualization. CAC coronary artery calcium, CCTA computed tomography (CT) coronary angiography.

**Table 1:** Overview of Groups of Topics With Example Text

Group	Topics	Discussions (#)	Mean Sentiment	Description	Examples
1	3, 4, 5, 8, 15, 33, 36, 39, 40, 41, 44, 62	1102	−0.38	Indication for CAC	“All is normal. Next up will be a **calcium score** of my heart. Cardiologists can’t figure out why my heart rate is so high.” “They took x-rays, an ekg and a **calcium score** test to check for blockages, but the pain kept coming and I had to specifically ask for an echo to eliminate all cardiac possibilities. I’m glad I did, because that’s what finally showed the pericarditis.”
2	1, 11, 12, 24, 27, 31, 51, 53, 57, 61, 64, 69, 86, 91	992	−0.48	Interpretation of CAC	“If I was in your shoes with a **CAC score** of 0, would have opted for lifestyle changes and diet only.” “I was in a similar situation at your age, though perhaps not as diligent on my diet. Was debating whether to go on a statin therapy or not (been now for over 12 years). The decisive factor for me was the **coronary calcium** scan - if the score was zero - I would have totally ignored statins. It wasn’t”
3	22, 37,42, 43, 45, 48, 54, 58, 59, 65, 66, 73, 74, 76, 78, 79, 81, 82, 85	662	−0.47	Downsides of CAC (Cost, Radiation)	“Get a cardiac **calcium score** done. It will tell you how much plaque if any is built up in your arteries and overall risk of heart attack. It’s not covered by insurance though and runs about $120–130. Small price to pay for the peace of mind though.” “Not sure you want to be doing a scan for **coronary calcium** too often given the radiation exposure involved. Definitely something to discuss with your doctor”
4	7, 14, 19, 23, 38, 77	500	−0.48	CAC and lipid panels	“I recently got a CT scan **calcium score** to check for arterial plaque and the results were “zero…My cholesterol is 225, but I do not subscribe to the lipid hypothesis, so no statins for me.” “Your chol numbers aren’t horrible. I concur with the other person who mentioned getting an NMR and **coronary calcium** done. High probability you will have a large LDL particle size, and negligible calcium score…and the score will likely get better as time goes on.”
5	6, 9, 35, 75	363	−0.41	CAC evaluation experience	“I actually have a few different monitors for my heart rate now. Have had a stress test, echocardiogram, **calcium score** test, and a 14 day heart holter test. There appears to be nothing wrong with my heart, just that it beats slowly now compared to when I was smoking.” “No cardiologist told me to stop when symptoms go away. It took 9 months but eventually my heart got better. It was scarry I had to do the treadmil test, **heart scan** etc to figure out but the type of arrhythmia was not that dangerous. It felt like my heart was stopping but there was actually one extra beat.”
6	16, 25, 26, 47, 72	338	−0.43	Diet and CAC	“My husband went from full carnist to vegan 35 years ago. He’s 60 now. He had zero calcifications on his **heart scan** last year (his father died at 57 from stroke).” “I’ve been keto carnivore for the last 3 years, and my total score for **coronary calcium** went this year to ZERO…and my cholesterol went skyrocket”
7	2, 10	301	−0.51	CAC vs. CCTA	“I’m sorry, I was asking if the **calcium score** test showing zero is something to go by for likelihood of having a normal coronary CTA. Meaning if it’s zero, is it likely a blockage would show on the CTA. Of course, yes, things always change and test should be repeated where warranted.” “I don’t know the answer to your first question. Re #2 - yes you can have a **calcium score** of zero and still have an LAD occlusion. Not all plaque is calcified. In fact, non-calcified plaque is more unstable. This is an over-simplification, but calcium suggests some chronicity and therefore stability”
8	13, 18, 60, 70, 83, 88, 89	294	−0.54	CAC and cholesterol	“Cholesterol numbers aren’t the most accurate marker of potential heart disease. Your **coronary calcium score** is a more accurate marker of heart disease.” “…I was overweight, in denial Bc I wasn’t gigantic. I’ve been 10–15 pounds overweight for years. I’ve also got high cholesterol. I finally went to get a **coronary calcium** scan, at my doctor’s urging, and it turns out I’m in the early stages of heart disease. I never really considered myself fat but clearly my weight wasn’t healthy. So now I’m eating better, and working out again. And I’m already down about 5 lbs.”
9	21, 28, 46, 67	238	−0.47	Pregnancy and heart scans	“When my daughter was born she had little holes in her heart. PDA (Patent Duct Arteriosus) and PDF (Patent Duct Fibrosis). Even after 20 years I’ll never forget those terms. Because we lived in a small country, it was a year before we could get a second **heart scan** appointment.…On the day of her scan, the doctor gave her the all clear…” “I’m on Zofran and Celexa (citalopram), it’s a SSRI. I had a meeting with a geneticist and risks are very small if any. The only concern they may have is a small infraction of the heart not closing all the way so at 20–22 weeks I will see a neocardiologist to do a full **heart scan** of baby.”
10	20, 34, 49	202	−0.53	Treatment for CAC	“I’ve done my own research, and I’m convinced statins are the spawn of satan and cholesterol is not bad…You can get a calcium **heart scan** for little money. It will give you a good idea of any plaque building in arterial walls.” “the calcification thing is hilarious because when it was shown. that statins increase calcified plaque they suddenly reasoned “it must be good” that it’s doing so, despite all the evidence showing a **calcium score** of 0 as being the most predictive of 10-year MACE risk”
11	29, 30, 50	196	−0.53	Heart scans and health-system issues	“They’ve all confirmed the UK has awful wait times. This is where Americans go wrong. We have long wait times ***for things that are non-dangerous and usually elective.***When Americans hear “long wait times” they seem to jump to the assumption that you have to wait 3 years to get cancer results back or a **heart scan**, which couldn’t be further from the truth.” “Get a **heart scan** people. 50 bucks without insurance. Could save your life!”
12	17, 52, 63, 90	175	−0.45	Pets and heart scans	“Paws crossed for Zoey! I had a cat (also a tux) years ago with a heart murmur and she just had to take a pill daily and have an annual **heart scan**. Other than that, she was all good!” “Personally I wouldn’t buy from a breeder who breeds from cats who have tested positive for a HCM gene, even if the kittens aren’t for breeding. Also with HCM a cat can test N/N and still have HCM as it’s a multifactorial genetic disease and not all genes have been identified. Best way is for the parents to have had a **heart scan**, however most breeders don’t do this as it’s expensive. But having a cat with HCM will cause them to likely doe before 2 years old and cost you lots of money. Buy from a breeder who takes it seriously.”
13	55, 56, 68, 71, 80	150	−0.37	Celebrities with CAC	“[Trump] is a hairs-breath from clinically obese and has a heart **calcium score** of 133, but is in “excellent health”? I mean, he’s not on death’s door, but that’s not “excellent” health by any stretch of the imagination.” “Tim Russert who died of a heart attack while having a normal blood panel. “On the negative side, Mr. Russert had low HDL, the protective cholesterol, and high triglycerides. He was quite overweight; a waist >40 inches in men increases heart risk. A CT scan of his coronary arteries in 1998 gave a **calcium score** of 210, indicating artery disease—healthy arteries do not have calcium deposits—and a moderate to high risk of a heart attack.”
14	32, 84, 87	93	−0.84	Recommending CAC	“Did you have a **coronary calcium heart scan**? If not and still concerned about your heart, look into this test. It may offer some reassurance.” “And you can now measure this directly with a **calcium score**. It’ll straight up tell you if there’s blockage in your heart.”

Our AI-enabled analysis of public perceptions of CAC testing demonstrates how well our previously described algorithm for topic modeling generalizes to another clinical domain^8^. A powerful aspect of our pipeline is leveraging techniques in unsupervised machine learning that obviate the need for topic prespecification, which allows discovery of previously unexpected ideas (e.g., non-evidence-based use of CAC). Such topic modeling analyses can also provide clinical insights that may be further explored to test generated hypotheses. By harnessing the power of AI on pre-existing datasets, we demonstrate a fast, inexpensive method of gathering public opinions that would otherwise require time- and finance-intensive clinical registries and user surveys to collect. Through this efficient extraction and interpretation of large volumes of social media data, AI also offers the ability to continuously evaluate public sentiment over time, monitor for emerging topics, and stream clinical insights to key stakeholders that could impact clinical care.

Our study revealed several noteworthy insights about public perceptions around CAC testing. First, CAC testing had a strong impact on therapeutic decision-making. Many discussions emphasized the power of a CAC score of zero as way of de-risking individuals and avoiding statin therapy. While a CAC-based de-escalation strategy is supported by practice guidelines, the presence of other risk-enhancing lifestyle or clinical factors (e.g., diabetes) may affect these decisions^10^. Conversely, many discussions where a non-zero CAC was noted demonstrated how these findings helped motivate lifestyle changes. Ultimately, CAC interpretation is nuanced, and our study highlights that public discussions around interpretation of CAC results may not always be guideline-concordant, underscoring the need for patient and clinician shared-decision making.

Second, there were several discussions surrounding non-evidence-based uses of CAC testing, including for evaluation of patients with cardiac symptoms, such as chest pain and palpitations. This may be discordant with current clinical guidelines, which endorse the use of CAC testing in primary prevention among asymptomatic patients, particularly those with intermediate ASCVD risk^3^. Many discussions also misattributed the negative predictive value of a CAC scan to evaluate non-specific symptoms typically not related to ASCVD risk assessment, which may further misrepresent the current indications for CAC to the public. Future work may focus on evaluating the dynamics of how such misinformation can be amplified in social media frameworks and ultimately help determine optimal strategies for containing their spread.

Third, we identified discussions regarding the disadvantages of CAC testing, including out-of-pocket costs due to lack of insurance coverage and radiation exposure. However, many individuals still found value in CAC testing despite costs and radiation. The cost-effectiveness of CAC has been reported elsewhere in the literature^11^. Although the radiation risk associated with CAC testing is minimal, similar to ambient radiation from living in large cities^12^, our work identified that patients may be concerned about this risk when deciding to pursue CAC testing.

Finally, we found that the sentiment around CAC-related discussions was mostly neutral-to-negative. This is consistent with prior studies evaluating healthcare discussions on Reddit, which identify a negative tone and expressions of sadness, fear, and anger that is believed to reflect the underlying patient experience in a complex healthcare environment^13^. This negativity bias is well reported in the media and can impact health outcomes^14^, suggesting the importance of public health efforts to moderate misinformation^15^.

This study should be interpreted in context of its limitations. Discussions in this study reflect views of Reddit users, who have historically been younger and may not be broadly representative of patients at high risk of ASCVD^16^; however, CAC testing is most appropriate for lower and intermediate risk individuals. While a variety of search terms were used, this dataset may not capture all CAC-related discussions on Reddit if individuals use other terms to refer to CAC. Clustering techniques we employed may reflect linguistic concordance to determine similarity rather than clinical concordance, which may lead to seemingly redundant topics and groups. This limitation highlights how AI can augment, but not replace, researchers in analyzing large datasets, and opens the door to consider how more advanced NLP techniques, like large language models, can improve this pipeline.

In this AI-enabled qualitative study of discussions on Reddit, we identified contemporary public perceptions and sentiments around CAC, which included the impact of CAC on therapeutic decision-making, non-evidence-based use of CAC testing, and the perceived downsides of CAC testing. The themes uncovered from this study highlight potential areas of patient concern and misinformation that can be addressed to improve shared decision-making around ASCVD management, improve statin adherence rates, and reduce ASCVD morbidity and mortality.

## Methods

### Dataset

Reddit (www.reddit.com) was used as the data source for this study^17^. It is composed of communities called ‘subreddits’ which are prefixed by “r/” and are focused on specific topics (e.g., r/AskDoctors, r/WorldNews, r/Keto). Users may interact with the platform by creating a “post” to initiate a new discussion thread and by commenting on other users’ posts as part of discussions (“comments”). Most subreddits, including all posts and comments contained within them, are openly accessible and visible without having to create a Reddit user account.

To create a list of CAC-related discussions from Reddit, an Application Programming Interface (API) called PushShift was used to search all the posts and comments on Reddit for case-insensitive matching on the following commonly used terms for CAC scans: “coronary artery calcium”, “coronary calcium”, “cac score”, “calcium score”, and “heart scan”^7,18^.

This study was deemed exempt from ethical review since it did not involve human subjects as defined in 45 United States’ Code of Federal Regulations (CFR) 46.102(f) or 21 CFR 50.3(g).

### Data analysis

Details around topic modeling and sentiment analysis in this paper are described elsewhere^8^. Briefly, after preprocessing, discussions are embedded into a numerical representation using a pretrained, sentence-level Bidirectional Encoder Representations from Transformers (BERT) model called all-MiniLM-L6-v2^19^, which has been trained on over 600 million Reddit posts and a dataset containing over 12 million papers from medical journals. This embedding was then simplified into a smaller representation using the Uniform Mapping Approximation and Projection algorithm to improve clustering performance into topics using Spectral Clustering. Since topics may be similar in content but be differentiated by other embedded features from the model (e.g., linguistic style, tone), a subsequent clustering analysis was performed to find overarching themes of discussion (“groups”). The number of topics and groups were automatically determined based on optimizing the Silhouette Coefficient and Davies-Bouldin Index, which are mathematical measures of how similar discussions are within a cluster relative to how similar those discussions are to those in other clusters. A separate BERT model, RoBERTa, pretrained on social media posts, was used to classify sentiment (i.e., “positive”, “neutral’, or “negative” classification of text).

### Reporting summary

Further information on research design is available in the Nature Research Reporting Summary linked to this article.

### Supplementary information

Supplemental Material

Reporting Summary

## Data Availability

The data used in this manuscript are available at https://github.com/sssomani/cac_reddit.

## Code availability

The code used in this manuscript is available at https://github.com/sssomani/cac_reddit.
