# Supplementary material for: Contemporary attitudes and beliefs on coronary artery calcium from social media using artificial intelligence
Source: NPJ Digit Med. 2024 Mar 30;7:83. doi: 10.1038/s41746-024-01077-w (PMC10981728; doi:10.1038/s41746-024-01077-w)

**Supplementary Figure 1. CAC-Related Posts and Comments Over Time**. This graph shows the percentage of all CAC-related discussions submitted each year, from 2008 to 2022.


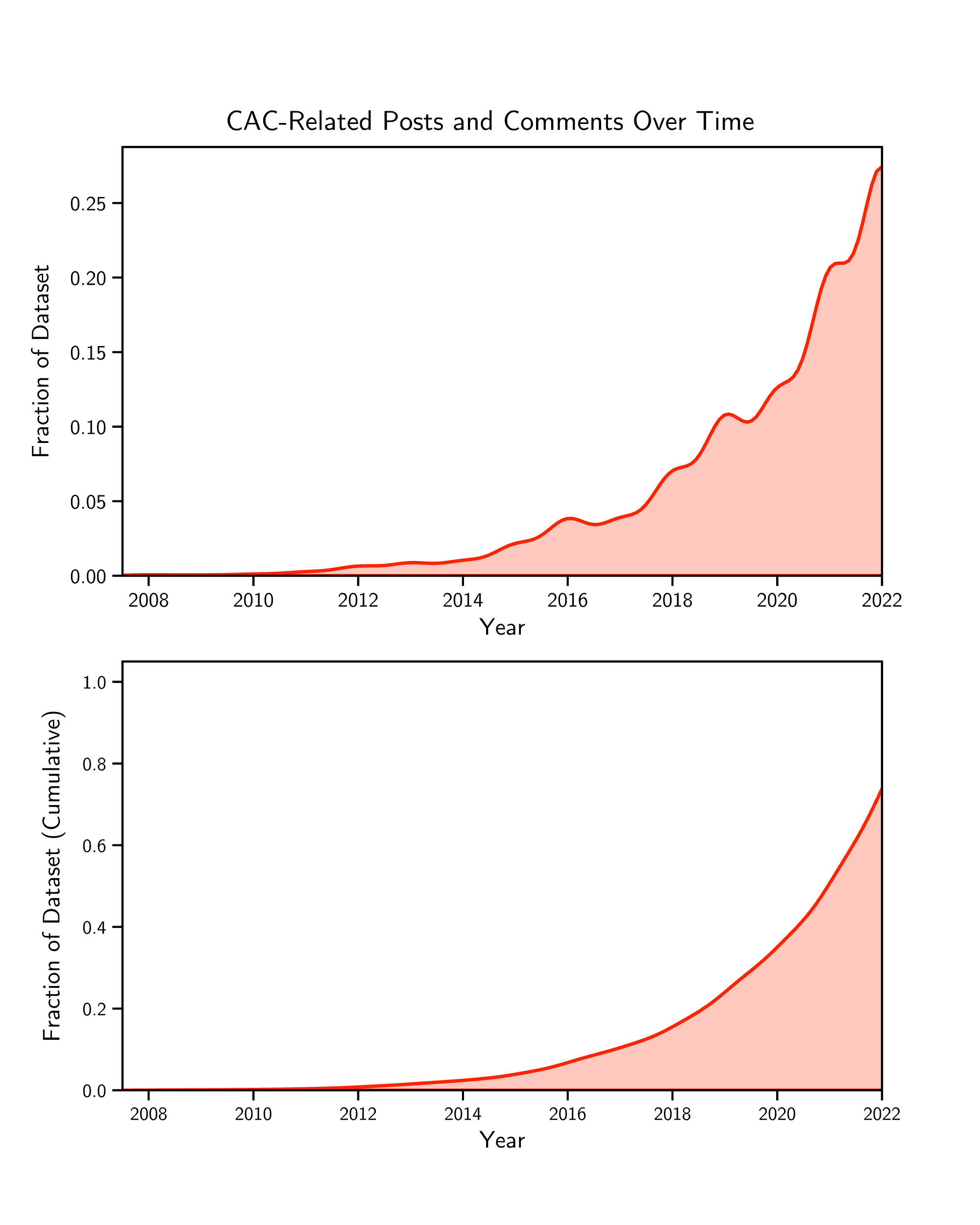


**Supplementary Figure 2. Mean Annual Discussion Sentiment**. This graph represents the mean sentiment of all discussion by year.


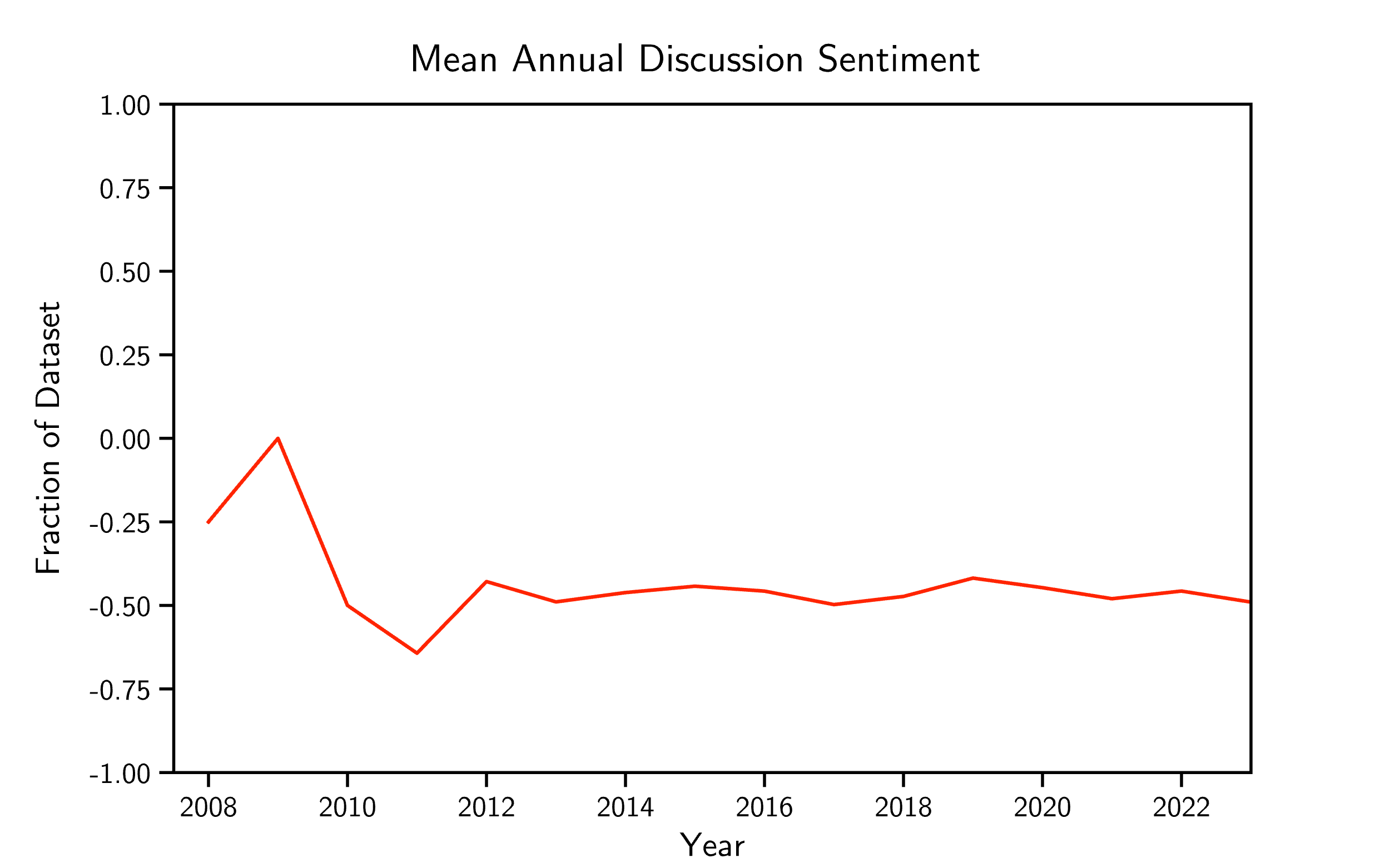

Supplement: Supplementary file 1 — Supplemental Material [file 41746_2024_1077_MOESM1_ESM.docx]
